# Navigating uncertainty in museum workflows: genomic data mining and curation of the Diptera collections hosted at RMCA

**DOI:** 10.3897/BDJ.13.e157274

**Published:** 2025-08-12

**Authors:** Lore Esselens, Pia Addison, Jacqueline Bakengesa, Luis Bota, Laura Canhanga, Domingos Cugala, Beatriz Daniel, Marc De Meyer, Hélène Delatte, Jean-Marc Herpers, Kurt Jordaens, Sija Kabota, Abdul Kudra, Ramadhani Majubwa, Aruna Manrakhan, Mirene Mussumbe, Maulid Mwatawala, Franck Theeten, Didier Van den Spiegel, Sam Vanbergen, Carl Vangestel, Massimiliano Virgilio

**Affiliations:** 1 Royal Museum for Central Africa, Tervuren, Belgium Royal Museum for Central Africa Tervuren Belgium; 2 University of Stellenbosch, Stellenbosch, South Africa University of Stellenbosch Stellenbosch South Africa; 3 Sokoine University of Agriculture, Morogoro, Tanzania Sokoine University of Agriculture Morogoro Tanzania; 4 The University of Dodoma, Dodoma, Tanzania The University of Dodoma Dodoma Tanzania; 5 National Fruit Fly Laboratory, Chimoio, Mozambique National Fruit Fly Laboratory Chimoio Mozambique; 6 Eduardo Mondlane University, Maputo, Mozambique Eduardo Mondlane University Maputo Mozambique; 7 Centre of Excellence in Agri-Food Systems and Nutrition, Maputo, Mozambique Centre of Excellence in Agri-Food Systems and Nutrition Maputo Mozambique; 8 Centre de Coopération Internationale en Recherche Agronomique pour le Développement, La Réunion, France Centre de Coopération Internationale en Recherche Agronomique pour le Développement La Réunion France; 9 Royal Belgian Institute of Natural Sciences, Brussels, Belgium Royal Belgian Institute of Natural Sciences Brussels Belgium; 10 Citrus Research International, Nelspruit, South Africa Citrus Research International Nelspruit South Africa; 11 University of Leuven, Leuven, Belgium University of Leuven Leuven Belgium; 12 University of Ghent, Ghent, Belgium University of Ghent Ghent Belgium

**Keywords:** natural history collections, museomics, whole genome sequencing, true fruit flies, hoverflies

## Abstract

As part of its extensive Diptera holdings, the Royal Museum for Central Africa (RMCA) houses over 100,000 specimens of Tephritidae and Syrphidae, which represent a critical resource for taxonomic and systematic research. Here, we present a feasibility study evaluating streamlined workflows for genomic data mining and archiving in museum collections. We analysed DNA yield, quality and sequencing performance from more than 1,400 insect vouchers and found few predictable trends, reflecting the nature of heterogeneous and skewed groups of samples collected under largely unknown field conditions. Regardless, our results show that Illumina short read whole genome sequencing can work well even with degraded insect material. In this context, routine short-read sequencing offers a practical first step for genomic data mining, particularly for large collections. It enables us to reserve more complex and resource-intensive methods for the subset of samples that fail initial sequencing (7% of specimens, in our case). As an outcome of this work, RMCA’s archiving system has been adapted to integrate genomic data and metadata alongside traditional specimen records. We argue that genomic data should be treated as an integral component of collection management, enhancing scientific value, supporting long term preservation and improving traceability of genetic resources in natural history collections.

## Introduction

Specimens from natural history collections offer unique taxonomic, evolutionary and ecological research opportunities, particularly for rare or extinct taxa or for species which are difficult to collect from the field (Nakahama 2020). In this context, the insect collections of the Royal Museum for Central Africa in Belgium (RMCA), represent historically important repositories of specimens collected during scientific expeditions and research projects in the Afrotropical Region (Sub-Saharan Africa) over the last 150 years. The collections are especially rich in specimens from the Democratic Republic of Congo, Burundi and Rwanda, primarily from the first half of last century, as well as other parts of Sub-Saharan Africa, with increased geographic coverage from the second half of the century onwards. In recent decades, the RMCA's policies regarding sample collection in the Afrotropical Region have undergone significant changes. These shifts were influenced by the growing international debate on decolonising scientific research (Springer Nature 2022), which gained momentum during the Museum's renovation in 2013-2018, as well as by the implementation of the Nagoya Protocol on Access and Benefit-Sharing (ABS, Secretariat Biological Diversity (2011)). The Nagoya Protocol, which entered into force in 2011, provides a legal framework to ensure fair and equitable sharing of benefits derived from genetic resources, thereby promoting ethical collaborations amongst global institutions. While Belgium signed the Protocol in 2011, it only ratified it and became a Party in 2016, which formally marked the beginning of its legal obligations under the treaty. In response, the RMCA has placed increasing emphasis on equitable partnerships and compliance with international agreements, either by adhering directly to the Nagoya Protocol or by strictly following its principles in African countries where it has not yet been implemented.

While substantial efforts have been made to valorise the RMCA collections through the digitisation of specimen images (see Acknowledgements), comparatively little attention has been given to unlocking the potential of the genetic resources embedded within these collections and making them accessible for scientific research. The continuous advances in genomic technologies are rapidly expanding the possibilities for accessing and analysing historical specimens. Methodological approaches to recover genomic data from specimens in natural history collections, once considered beyond the technological capabilities of most institutions (Colella et al. 2020) are becoming increasingly cost and time effective (Ferrari et al. 2023). In this respect, a growing number of dedicated -omics protocols for degraded DNA from museum vouchers have been developed to mine genomic data from museum collections (Guschanski et al. 2013, Knyshov et al. 2019a, Knyshov et al. 2019b, Mullin et al. 2022). A particular challenge arises with suboptimal samples, which, in the context of this study, refer to specimens typically ranging from a few years to several decades in age. In addition to age, the way in which these specimens were preserved, such as storage temperature and preservation medium, also significantly affects DNA quality and downstream analyses (Carter and Walker 1999, Ferrari et al. 2023. The suboptimal samples are neither as high-quality as freshly collected and properly preserved material nor as degraded as truly historical specimens. Researchers working with such material are in a limbo and must decide between standard, cost- and time-efficient laboratory protocols, which may be inadequate for degraded DNA and more complex, time-consuming and often more expensive procedures tailored to highly degraded samples.

Ready-to-use genomic data recovered from natural history collections hold significant potential for both fundamental and applied research. This is especially relevant for our insect holdings, which include extensive collections of Afrotropical Tephritidae (true fruit flies) and Syrphidae (hoverflies). These collections, comprising over 100,000 specimens, are the focus of active taxonomic and phylogenetic research (De Meyer et al. 2016, Delatte et al. 2019, De Meyer et al. 2020a, De Meyer et al. 2020b, De Meyer et al. 2024, Deschepper et al. 2024a, Deschepper et al. 2024b). The samples span a collection period of up to 120 years, with a notable increase in collecting activity over the past 25 years. They include both older specimens and more recently collected material, preserved either as dry-pinned specimens or in absolute ethanol at −20 °C or −80 °C. A subset of these specimens has been whole genome sequenced as part of multiple research projects (see Acknowledgements), either through non-destructive processing of the entire voucher or by processing selected parts, such as legs.

Here, we report the results of a feasibility study that aimed to: (a) develop and implement standardised workflows for Illumina short-read whole genome sequencing (WGS) of Diptera in the RMCA collections; (b) establish a sustainable archiving strategy to enhance the long-term usability of the resulting genomic datasets; and (c) integrate these genomic data into the collection management system of RMCA to ensure linkage of sequence information with collection specimen. We believe this pilot might serve as a useful starting point for comparable natural history collections looking to routinely mine and curate genomic data.

## Materials and methods

### Taxon sampling

**Figure 1.**
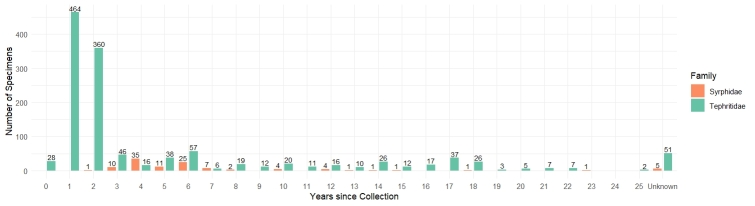
Number of specimens per year since collection (1,405 Tephritidae and Syrphidae processed, including 56 with missing collection year and indicated as “Unknown”).

This study refers to a selection of DNA extracts from 1,405 insect vouchers that were collected between 1997 and 2022 in 54 countries across Africa (925 specimens), Europe (367), Asia (83) and America (30). The dataset is strongly skewed towards Tephritidae (1,296 specimens, representing 79 species from the genera *Bactrocera*, *Ceratitis* and *Dacus*) with a smaller representation of Syrphidae (109 specimens from the genera *Eristalinus* and *Melanostoma*) (Fig. 1; Suppl. material 1). This bias reflects the research activities of the RMCA entomology section, which, over the past few decades, have primarily focused on tephritid fruit flies. In contrast, research on Syrphidae is more recent and, in this study, they were included as a secondary test group to assess the broader applicability of our laboratory and sequencing pipeline across different dipteran families. Furthermore, the specimens from the two groups differ in age distribution, with the Tephritidae showing a more skewed pattern as approximately 70% were collected within the last four years, while the Syrphidae mainly originate from a narrower window between 2015 and 2019. While we fully acknowledge the limitations of this non-uniform sampling, we consider it may offer insights into how specimen age influences high throughput sequencing across a range of realistic conditions.

### DNA extraction and whole genome sequencing

The performance of widely used commercial DNA extraction kits (QIAGEN, Suppl. material 2) was preliminarily and semi-quantitatively explored on a subsample of insect vouchers. DNA yields were estimated on three to eight specimens from five species of the target families from seven collection events between 2008 and 2016. This timeframe was selected as deemed to be representative of samples processed in the main experiment (see below). These included three tephritid species, *Zeugodacuscucurbitae*, *Bactroceradorsalis* and *Dacusbivittatus*, as well as two Syrphidae species, *Eumerus* sp. and *Ischiodonaegyptius*. Specimens were pinned and preserved at room temperature (*I.aegyptius*) or stored in absolute ethanol at -20°C (all other species). Digestion in lysis buffers was implemented on 48 entire specimens or on a single foreleg per specimen to test if using less material to be less destructive resulted in similar DNA yields (two negative controls with Milli-Q water were also included). The DNA lysates obtained from these samples were divided into four aliquots. Each aliquot was then purified using spin columns from one of the four DNA extraction kits (Suppl. material 2) following the manufacturer’s instructions. The concentration of each DNA extract was measured using a Qubit 3 fluorometer (HS DNA Kit, Thermo Fisher Scientific) and the total amount of DNA was inferred from the final elution volume (100 µl). The semi-quantitative comparison of results showed that the kits did not yield major differences in terms of DNA quantity, with the possible exclusion of the MiniElute kit on whole bodies, which had a lower performance (Suppl. material 3). Based on these results, we decided to rely on the kit with the lowest price, i.e. the DNeasy Blood & Tissue Kit (QIAGEN 69506), for the routine processing of specimens.

The suitability of museum Diptera for Illumina short-read WGS was then assessed in a larger experiment involving 1,405 specimens collected between 1997 and 2022. This dataset included taxonomically and temporally diverse specimens, 94.2% being stored in absolute ethanol in freezers at -20°C and -80°C, with the remainder preserved as pinned specimens and dried DNA extracts at room temperature. In all cases, the DNA was extracted using the DNeasy Blood & Tissue Kit (QIAGEN) as per manufacturer’s instructions. The DNA extractions were implemented on mostly whole vouchers (99.4%) and, for a very minor part, on insect abdomens or legs. DNA was eluted in volumes ranging from 60-120 µl.

DNA quantity and quality, as well as the HTS performance, were evaluated following DNA extraction. For each sample, the DNA quantity was measured as the total DNA yield per specimen calculated by the DNA concentration, as quantified by using a Qubit 4 fluorometer (HS DNA Kit, Thermo Fisher Scientific) and the elution volume. The DNA quality was measured as the distribution of DNA fragment size by the proportion of the DNA molarity of fragments shorter than 350 bp over the total DNA molarity and DNA purity by the absorbance ratios. The distribution of DNA fragment sizes was quantified on a subset of 902 samples by using a fragment analyser (Advanced Analytical) in combination with the DNF-930 dsDNA Reagent Kit, which covers a range of 75 bp to 200,000 bp (Genomics Core, Leuven, Belgium). The ProSize Data Analysis Software v.4.0.1.4 (Agilent Technologies) was used to estimate the DNA molarity (nmol/l) of each sample and the proportion of the DNA molarity of fragments shorter than 350 bp over the total DNA molarity. DNA purity was estimated by measuring the absorbance ratios A260/280 and A260/230 with an Implen NanoPhotometer N60 Touch on a subset of 720 samples, using two different absorbance ratios aimed at detecting DNA contamination from different sources such as proteins, phenol, carbohydrates, lipids and salts (*Lucena-Aguilar et al. 2016*). Average absorbances were calculated from three replicated measures to account for measure variability and increased accuracy. Following Lucena-Aguilar et al. (2016), DNA was semi-quantitatively categorised as “pure” (1.7 < A260/280 < 2.0 or 1.8 < A260/230 < 2.2) or “contaminated” (absorbance ratios outside these ranges). The proportion of “pure” DNA samples over the total number of samples was calculated per collection year.

Samples that showed detectable DNA yield (≥ 0.0001 µg) were subjected to Illumina short-read WGS at 10x coverage through the NEBNext Ultra II DNA Library Preparation Kit (New England Biolabs, USA) implemented by Berry Genomics (1,011 DNA samples) or Novogene with standard (178 DNA samples) or low-input library preparation (126 DNA samples), where the genomic DNA was randomly sheared into short fragments, end repaired, A-tailed and ligated with Illumina adapters. Indexing PCR was then performed using Illumina-compatible indexing primers and the libraries were pooled, based on their effective concentrations and expected data output, ensuring optimal sequencing capacity utilisation. The Illumina NovaSeq platform generated 150 bp paired-end reads with a targeted 6 Gb raw data output per sample. To further evaluate the performance of additional steps along the HTS pipeline we then considered: (a) the proportion of high-quality reads (i.e. with a Phred Q-score higher than 30) and (b) the proportion of reads mapped to a reference genome of *Drosophilamelanogaste* (GenBank accession no. GCA_029775095). While (a) provided a first estimate of HTS performance, (b) was used to estimate the proportion of target sequences successfully obtained, as the alignment to a closely-related dipteran reference genome excluded most of the non-dipteran contaminant reads. Raw reads were trimmed using the *fastp* tool (*Chen et al. 2018*) and mapped to the *D.melanogaster* reference genome using the *bwa-mem* command from the burrows-wheeler aligner tool (Li and Durbin 2009).

### Data analysis

Relationships amongst voucher age, DNA quality and HTS performance were assessed using general(ised) linear models. As sampling was structured differently for the two families, leading to differences in the distribution of samples over time, models were fitted separately for Tephritidae and Syrphidae and the results were compared descriptively to identify patterns. Furthermore, as they represented the vast majority of samples, only specimens preserved in ethanol were included in the analyses. Voucher age was included as the continuous independent variable. Dependent variables included: (a) DNA quantity, (b) proportion of the DNA molarity of short fragments (< 350bp), (c) proportion of pure DNA samples (according to absorbance ratios A260/280 and A260/230), (d) proportion of high quality reads and (e) proportion of reads aligned to the reference genome of *D.melanogaster*. Trends for all variables, except absorbance ratios, were assessed using linear models (LM) with significance tested by F-statistics. Absorbance ratios were modelled using a generalised linear model (GLM) with a binomial error distribution and significance was tested by Chi-square. Variables were initially assessed for normality and homoscedasticity. Model assumptions, including normality and homoscedasticity, were checked using residual diagnostics and Q–Q plots and, where heteroscedasticity was detected, weighted least squares (WLS) approaches were employed. Voucher age was included as fixed effect (*Zuur et al. 2013*). All models were fitted using the *lme4* package (version 1.1-37) in *R* (version 4.4.3). Visualisation of model fits and observed data was performed using the *ggplot2* package (version 3.5.2) in R, employing bar plots of observed proportions combined with regression lines and confidence ribbons derived from the models to effectively illustrate the relationships between voucher age and DNA quality/HTS performance metrics for each family.

### Archiving of DNA samples and genomic data

Gen Tegra-DNA 2D barcoded vials with a silica matrix (https://gentegra.com/gentegra-dna-2/) were used for long-term archiving at room temperature of the extracted DNA. A subset of 792 images from 396 vouchers were generated using a focus stacking imaging system (Zerene Stacker software), composed of commercial photographic equipment (Canon DSLR camera and Canon microlens) (*Brecko et al. 2014*). Images from 292 *Dacus* and *Ceratitis* (Diptera, Tephritidae) specimens were also selected for publication on the https://fruitflies.africamuseum.be/ website. As part of a larger effort to digitise the RMCA collections, we relied on the DaRWIN collection management system to archive DNA samples and genomic data (Adam et al. 2019).

## Results

**Figure 2.**
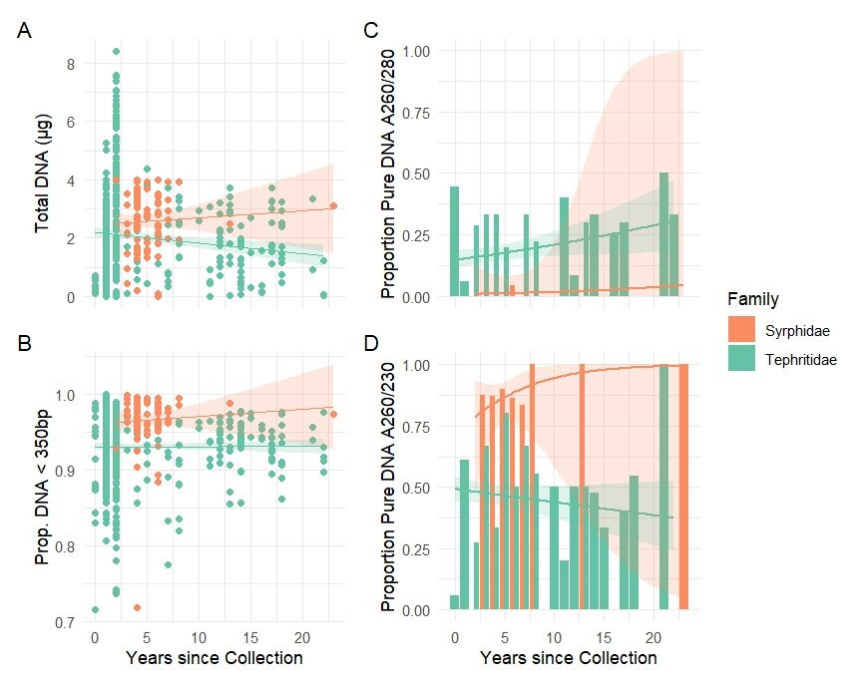
DNA quantity and quality (linear regression lines are indicated for Syrphidae and Tephritidae). Relationships between years since collection and (**A**) total DNA recovered per specimen (µg), (**B**) proportion of the DNA molarity of short DNA fragments (< 350 bp), (**C**) proportion of “pure” DNA of A260/280 and (**D**) proportion of “pure” DNA of A260/230.

The total DNA yield using four commercial DNA extraction kits from QIAGEN on the same specimen ranged from 0.06 µg to 0.15 µg, with an average of 0.65 µg (± 0.53 µg) for whole bodies and from 0.001 µg to 0.022 µg for legs, with an average of 0.006 µg (± 0.006 µg). The use of the DNeasy Blood & Tissue Kit (QIAGEN) on 1,405 insect vouchers produced yields between 0.00 µg and 8.83 µg, with an average of 2.20 µg (± 1.53 µg). Total DNA yield was significantly affected by voucher age in tephritid specimens (*F*_1,1174_ = 45.09, *p* < 0.001) (Table 1, Suppl. material 4), with more recently collected specimens yielding higher DNA amounts. In contrast, no significant relationship between voucher age and DNA yield was found in syrphids (*F*_1,85_ = 0.38, *p* = 0.54) (Fig. 2A, Table 1, Suppl. material 4).

The proportion of the DNA molarity of fragments shorter than 350 bp per sample varied between 0.542 and 1 with an average of 0.93 (± 0.059) and 77.9% of samples having a proportion of short DNA fragments higher than 0.90. Voucher age had no significant effect on the proportion of short DNA fragments in either group (*F*_1,738_ = 0.34, *p* = 0.56 for Tephritidae; *F*_1,88_ = 0.24, *p* = 0.62 for Syrphidae) (Fig. 2B, Table 1, Suppl. material 4).

According to the A260/280 absorbance ratio, 'pure' DNA was present in only 16% of the samples (116 out of 720), while the A260/230 absorbance ratio indicated that 49% of the samples yielded “pure” DNA (351 out of 720). In Tephritidae, the proportion of pure DNA based on A260/280 decreased significantly in more recent vouchers (*LR χ*² = 53.04, *df* = 4, *p* < 0.001), while no significant effect was found in Syrphidae (*LR χ*² = 0.072, *df* = 1, *p* = 0.79) (Fig. 2C, Table 1, Suppl. material 4). Similar trends were seen for the A260/230 ratio, with purity decreasing over time in Tephritidae (*LR χ*² = 42.13, *df* = 4, *p* < 0.001), but no significant changes in Syrphidae (*LR χ*² = 0.22, *df* = 1, *p* = 0.64) (Fig. 2D, Table 1, Suppl. material 4).

Out of 1,405 DNA extracts, 1,315 (93.6%) contained measurable amounts of DNA and were subjected to genomic library preparation and sequencing. Of these, 1,305 samples (99.2%) were successfully sequenced (i.e. provided WGS data of approximately 10x coverage). Overall, 92.9% of all samples processed in this study were successfully sequenced and produced genomic data for downstream genomic analyses, including population genomics, with some of these samples also contributing to the study by Deschepper et al. (2024a). The average cost for sequencing these samples, including those that failed and those for which sequencing was outsourced to two companies, corresponded to €77.25 ± €39.01. This average reflects two pricing schemes: the majority of samples were sequenced at €73 per sample by one provider, while a subset was processed by a second provider at €128 per sample. For particularly low-input DNA extracts requiring specialised library preparation, the cost increased to €148 per sample. In addition to sequencing, the cost of DNA extraction using the DNeasy Blood & Tissue Kit (QIAGEN) was €5.70 per sample (excluding personnel costs).

**Figure 3.**
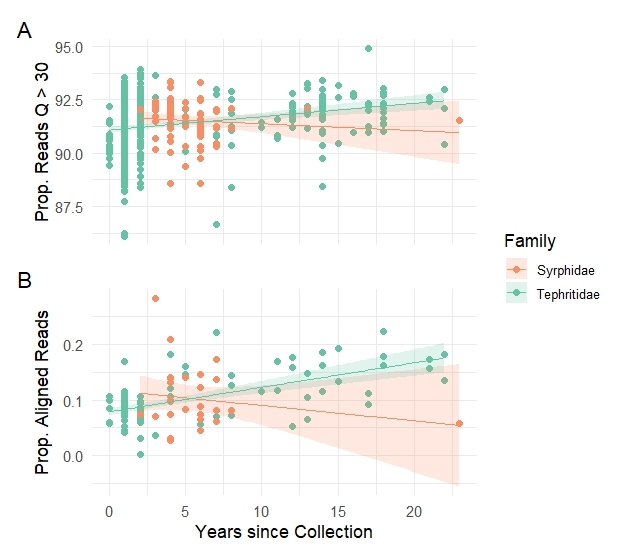
Performance of whole genome sequencing (linear regression lines are indicated for Syrphidae and Tephritidae). Relationships between years since collection and (**A**) proportion of high-quality reads (Q > 30) and (**B**) proportion of reads aligned to the reference genome of *Drosophilamelanogaster* (GCA_029775095.1).

The proportion of high-quality reads (i.e. with Phred quality score Q > 30) ranged from 0.86 to 0.96 in 1,305 samples, with an average of 0.92 (± 0.15). Voucher age significantly affected the proportion of high-quality reads in Tephritidae, with newer specimens showing a lower proportion (*F*_1,1078_ = 11.98, *p* < 0.001). No significant effect of age was found in Syrphidae (*F*_1,88_ = 0.53, *p* = 0.47) (Fig. 3A; Table 1; Suppl. material 4). The proportion of reads aligned to the *D.melanogaster* reference genome ranged from 0.0025 to 0.28, with an average of 0.10 (± 0.047). In Tephritidae, this proportion decreased significantly in more recent vouchers (*F*_1,106_ = 38.80, *p* < 0.001), while no significant differences were found in Syrphidae (*F*_1,26_ = 0.87, *p* = 0.36) (Fig. 3B; Table 1, Suppl. material 4).

GenTegra DNA 2D barcoded vials with a silica matrix (https://gentegra.com/gentegra-dna-2/) were used for long-term archiving of the extracted DNA at room temperature. The tube barcode and position were linked to the DaRWIN collection voucher (Adam et al. 2019). A numeric lab book is now available to collect specimen and laboratory information and automate data export to DaRWIN, via Visual Basic macros. An analysis study is ongoing to link DaRWIN with the external Laboratory Information Management System. In addition, the digital images produced for a subset of samples are available as links to the DaRWIN collection vouchers. The backup and long-term storage of these data was ensured by the network-attached storage systems of RMCA.

## Discussion

This study focused on developing standardised wet-laboratory and bioinformatic pipelines for the efficient generation, preservation and integration of genomic data from the RMCA Diptera collections, with particular attention to their long-term incorporation into the collection management system. By anchoring genomic data to physical vouchers and curatorial metadata, we aimed to enhance the accessibility of museum-based genomic resources for scientific research, including applications in integrative taxonomy and insect systematics.

### Balancing cost and uncertainty in WGS for insect museum collections

One of the main constraints limiting the routine production of genomic data from museum collections has been the cost associated with library preparation and sequencing, especially when working with large numbers of specimens. For this reason, partial genome sequencing strategies, such as reduced representation libraries (Ewart et al. 2019) or mitochondrial genomics (Timmermans et al. 2015), have traditionally been preferred for large-scale applications. We opted for standardised, commercially available and cost-effective lab pipelines instead of specialised protocols for ancient DNA or museomics (Straube et al. 2021, Andreeva et al. 2022), as these are not designed for routine HTS despite their potential of producing better results (Gauthier et al. 2020, Orlando et al. 2021, Hawkins et al. 2022). Rapid advances in sequencing technologies are steadily driving down costs and, as a result, routine and large-scale WGS of voucher specimens is becoming an increasingly viable and valuable approach for unlocking the genomic potential of entire museum collections (Gillett et al. 2014, Malakasi et al. 2019, Strijk et al. 2020, Mullin et al. 2022).

Insect museum collections are often characterised by unbalanced sampling across taxa, time periods and preservation methods. In our study, this imbalance is compounded by largely unknown field sampling conditions for most specimens. These uncertainties hinder our ability to discern clear patterns of sample suitability for genomics and the difficulty of predicting DNA quality or sequencing success is likely endemic to insect museum collections. Such high variability typically warrants a cautious approach, involving an in-depth assessment of each sample’s DNA quality before applying targeted methods to resolve underlying degradation. However, the results of this study might offer a complementary perspective. Surprisingly and despite the relatively high proportion of suboptimal samples with highly fragmented or contaminated DNA, about 93% of our specimens were successfully sequenced using standard, cost- and time-effective protocols for DNA extraction, genomic library preparation and WGS. While we do not advocate indiscriminate sequencing of insect museum collections, our results show that Illumina short read WGS can work well even with degraded insect material. In this context, routine short-read sequencing offers a practical first step for genomic data mining, particularly for large collections. It enables us to reserve more complex and resource-intensive methods for the subset of samples that fail initial sequencing (which in our specific case represented 7% of specimens). This strategy could become increasingly viable as the costs of -omics technologies continue to decline. Its effectiveness may also improve as a growing number of high quality insect reference genomes (Deschepper et al. 2024b) allow for more accurate mapping and assembly of Illumina short read data.

### DNA quality prediction hindered by sparse sampling history and storage conditions

The parameters considered in this feasibility study for the Illumina short-read WGS of the Diptera collections of RMCA revealed marked differences between the two insect families. Syrphidae samples demonstrated greater stability in DNA quality and sequencing metrics over time. Specifically, voucher age did not significantly affect DNA yield, DNA fragmentation (proportion of short DNA fragments), DNA quality or sequencing success (proportion of high-quality reads and alignment rates) in Syrphidae specimens, indicating relatively consistent preservation quality across the sampling window. In contrast, the DNA quantity of the Tephritidae specimens increased significantly in more recently collected vouchers. However, DNA quality metrics in Tephritidae showed clear age-related declines: neither the proportion of pure DNA samples, based on absorbance ratios, nor the proportion of high-quality sequencing reads improved with age. Instead, more recent Tephritidae samples exhibited higher DNA fragmentation and a significant decrease in the proportion of high-quality reads and aligned sequences, suggesting increased degradation or other factors negatively affecting sequencing performance in younger specimens. These differences likely reflect the uneven sampling efforts carried out over the years across the two collections, underscoring the complex interplay of biological and environmental processes that influence the suitability of insect specimens for genomic research. Previous studies have shown that specimen size, tissue type and preservation methods significantly affect DNA quality and yield (Kistler et al. 2017, Brewer et al. 2019, Card et al. 2021, Andreeva et al. 2022, Mullin et al. 2022). Additionally, differences between specimens, taxon groups or museum collections are likely influenced by intrinsic features of each insect group as well as by the heterogeneous sampling, collection management or laboratory procedures implemented by different curators, taxonomists and molecular biologists employed across institutions.

In this articulated scenario, part of the differences in DNA yield, quality and contamination between Syrphidae and Tephritidae may be attributed to how the specimens were collected and stored in the field. As is often the case with specimens from natural history collections, detailed information on collection and preservation procedures in the field is lacking for most of our samples, limiting our ability to systematically assess their impact. We do know, however, that nearly all Tephritidae and Syrphidae specimens were preserved in ethanol hours, days or even weeks after being trapped. Although detailed metadata are missing, general differences in field collection and preservation practices between the two groups are known and provide useful context for interpreting the results. While most Syrphidae were collected via hand netting and immediately preserved in absolute ethanol, Tephritidae were primarily collected using McPhail traps. These traps, specifically developed for monitoring true fruit flies in area-wide surveillance programmes, sterile insect technique operations, orchards and quarantine zones (FAO and IAEA 2018), contain an insecticide and a chemical lure to attract the target insects. They are typically left in the field for periods ranging from hours to several weeks before being emptied, depending on logistical constraints and the research objectives. Hence, if the traps are surveyed after long periods, the DNA of trapped specimens is more exposed to degrading environmental conditions, especially under (sub)tropical conditions (Fowler et al. 2024). Controlled laboratory trials and field evaluations conducted by Fowler et al. (2024), showed that high ambient temperatures and relative humidity significantly accelerate DNA degradation in tephritid flies, especially when exposure times are prolonged and specimens are not immediately preserved. These findings are consistent with previous studies reporting that elevated temperature and humidity can have detrimental effects on DNA quality in insects (Mandrioli 2008, Zimmermann et al. 2008), especially when collection protocols do not involve direct preservation in ethanol (Ballare et al. 2019). Similar concerns have been raised in recent field evaluations, which showed that the duration and type of trap exposure can significantly influence the suitability of Tephritidae for downstream molecular diagnostics (Brown et al. 2025). This aligns with broader evidence that the persistence of amplifiable DNA is highly sensitive to environmental conditions, particularly high humidity and rainfall, which accelerate degradation over time when specimens are not promptly preserved (Lee et al. 2019). Prolonged exposure under such conditions has repeatedly been shown to reduce DNA quality, as demonstrated in multiple studies highlighting the combined effects of temperature, humidity and exposure duration on DNA integrity (Lund and Dissing 2004, Stevens et al. 2011, Martoni et al. 2021, Butterwort et al. 2022). In addition, the temporal patterns identified in Tephritidae likely reflect heterogeneity arising from the distinct histories of specific sample groups. A fairly large group of Tephritidae collected in 2020 and 2021 was part of a single research project and yielded particularly degraded specimens (Fig. 1). Although these samples were collected recently, the lack of detailed information limits our understanding of their precise handling and storage conditions in the field. Based on what is known about typical collection practices in our research group, we can speculate that these specimens may have been subjected to prolonged, suboptimal conditions such as high temperature and humidity. This emphasises the critical role of field sampling protocols, including frequent servicing of traps and immediate storage in absolute ethanol. In this context, even limited metadata, such as the presence of a collection date or date range could help infer the likely collection method and exposure time, offering a valuable proxy for assessing preservation quality. More importantly, it highlights the challenges of reconstructing the collection history of samples collected under diverse field conditions that are often undocumented and beyond the curator’s control. Therefore, the inherently heterogeneous nature of natural history collections makes it challenging to predict consistent patterns in DNA yield, quality or sequencing performance across samples. As a result, the common expectation that more recent samples will consistently provide better genomic data may often be unmet.

### Integrated curation of genomic data

This pilot allowed integrating genomic data into DaRWIN, the open-source database platform developed by and currently in use at the Royal Belgian Institute of Natural Sciences (RBINS, https://darwin.naturalsciences.be/) and the Royal Museum for Central Africa (RMCA, https://darwinweb.africamuseum.be/). DaRWIN is built on PostgreSQL for data management and uses Symfony, a PHP framework, for its user interface and search engine. The platform’s source code is openly available on GitHub (https://github.com/naturalsciences/Darwin). The archiving system offers a public search engine and a back-end for scientific and curatorial collection management. The system represents taxonomic hierarchies with synonyms and allows taxonomic types to be flagged in the digital collection. It also enables the management of loans and the automatic labelling of physical specimens. It offers an importation pipeline for batches of data from tab-delimited or spreadsheet documents that checks and validates the taxonomic hierarchy of imported data and guarantees the alignment of the new data with its normalised relational model (taxa, collectors and collection locations).

RMCA and RBINS have adopted a common archiving technology, while maintaining separate collection management systems to support institution-specific workflows. The adoption of a shared archiving platform enhances compatibility and coordination across research institutes, given the close collaboration between RMCA and RBINS, two Belgian Federal Scientific Institutions supported by the same funding agency (the Belgian Science Policy), sharing laboratory infrastructure as well as a Joint Experimental Molecular Unit (JEMU; https://www.jemu.be/).

As a result of this study, the DaRWIN interface has been enhanced to integrate links to genomic data and metadata alongside traditional collection information. Specifically, the platform can now track the full chain of custody by linking each source specimen to its corresponding tissue sample and DNA voucher. These records are also connected to external resources, including genomic datasets archived on the RMCA servers. Metadata from these records are publicly accessible on this DaRWIN page. Genomic data are gradually made open access, for example, by providing GenBank accessions, following the publication of results from various research projects. Within the broader field of museomics, our work illustrates how a collection management system can be upgraded to support the archiving of genomic data and metadata. Treating genomic data not as ancillary, but as an integral component of collection management bridges advanced genomics and traditional curation of museum collections. Importantly, it also enhances the traceability of the genetic resources originating from natural history collections. By recording and publishing the full chain of custody from specimen to sequence, it promotes compliance with international agreements on sample properties and loans. In this context, we believe that the routine archiving and curation of genomic data would favour more reproducible, transparent and inclusive collection-based research.

In summary, our study shows that Illumina short-read WGS is a workable approach for large-scale sequencing of insect museum specimens using standardised and cost-effective protocols. We identified significant predictors of sequencing success, including taxon-specific effects and field collection methods, though their impact varied. Finally, by integrating genomic metadata into a curated database platform, we laid the groundwork for genomic traceability in museum collections. These results offer practical insights for future museomic initiatives and underscore the importance of metadata standardisation and curation in genomic research.

## Supplementary Material

AE8A4B35-7D3C-5EE0-822D-75BE14B82AEC 10.3897/BDJ.13.e157274.suppl1 Supplementary material 1 Species list Data type Table Brief description Species list of specimens processed in this study. File: oo_1349704.pdf https://binary.pensoft.net/file/1349704 Lore Esselens

03B49B08-D5C1-5B2D-B9F7-9E0BF3872697 10.3897/BDJ.13.e157274.suppl2 Supplementary material 2 DNA extraction kits Data type Table Brief description DNA extraction kits used for the preliminary comparisons. File: oo_1349707.pdf https://binary.pensoft.net/file/1349707 Lore Esselens

DDE94A3C-E8A6-53AA-A2FB-F1603273F9C2 10.3897/BDJ.13.e157274.suppl3 Supplementary material 3 DNA yields from DNA extraction protocols Data type Graph Brief description Total DNA yields from four DNA extraction protocols. File: oo_1314149.pdf https://binary.pensoft.net/file/1314149 Lore Esselens

6F3F9DE9-EE44-56FD-B72D-665AF747CF50 10.3897/BDJ.13.e157274.suppl4 Supplementary material 4 ANCOVA Data type Table Brief description Analysis of Covariance of the general(ised) linear models testing. File: oo_1369859.pdf https://binary.pensoft.net/file/1369859 Lore Esselens

## Figures and Tables

**Table 1.** Table 1: Results of general(ised) linear models testing the effect of voucher age (0–25 years) on six variables. Significance levels: * P < 0.05; ** P < 0.01; *** P < 0.001; n.s. non-significant. Detailed ANCOVA results are provided in Suppl. material 4.

**variables**	**Tephritidae**	**Syrphidae**
total DNA / voucher	***	n.s.
proportion of short DNA fragments (< 350bp)	n.s.	n.s.
absorbance ratio 260/280	***	n.s.
absorbance ratio 260/230	***	n.s.
proportion of quality reads (Q > 30)	***	n.s.
proportion of aligned reads	n.s.	n.s.
