# Supplementary material for: Navigating uncertainty in museum workflows: genomic data mining and curation of the Diptera collections hosted at RMCA
Source: Biodivers Data J. 2025 Aug 12;13:e157274. doi: 10.3897/BDJ.13.e157274 (PMC12365672; doi:10.3897/BDJ.13.e157274)
Supplement: Supplementary material 1 — Species list [file bdj-13-e157274-s001.pdf]

SM 1: Species list of specimens processed in this study.

Genus	Species	n
Bactrocera	*dorsalis*	146
Bactrocera	*latifrons*	7
Bactrocera	*mesomelas*	4
Bactrocera	*zonata*	106
Ceratitis	*capitata*	424
Ceratitis	*quilicii*	213
Ceratitis	*quilicii / rosa* *	4
Ceratitis	*rosa*	192
Dacus	*adustus*	3
Dacus	*africanus*	2
Dacus	*annulatus*	2
Dacus	*apostata*	1
Dacus	*arcuatus*	2
Dacus	*armatus*	6
Dacus	*aspilus*	1
Dacus	*bakingiliensis*	1
Dacus	*bivittatus*	19
Dacus	*brevistriga*	2
Dacus	*ceropegiae*	1
Dacus	*chiwira*	3
Dacus	*ciliatus*	6
Dacus	*congoensis*	1
Dacus	*demmerezi*	5
Dacus	*diastatus*	5
Dacus	*durbanensis*	4
Dacus	*eclipsis*	3
Dacus	*famona*	4
Dacus	*ficicola*	1
Dacus	*frontalis*	4
Dacus	*fuscinervis*	1
Dacus	*fuscovittatus*	2
Dacus	*humeralis*	22
Dacus	*hyalobasis*	4
Dacus	*kariba*	2
Dacus	*langi*	4
Dacus	*longistylus*	7
Dacus	*masaicus*	4
Dacus	*mediovittatus*	3
Dacus	*namibiensis*	2
Dacus	*pallidilatus*	2
Dacus	*pergulariae*	2
Dacus	*phloginus*	1
Dacus	*pulchralis*	1

Genus	Species	n
Dacus	*pullescens*	1
Dacus	*punctatifrons*	6
Dacus	*quilicii*	1
Dacus	*siliqualactis*	4
Dacus	*sphaeristicus*	2
Dacus	*stentor*	1
Dacus	*telfaireae*	3
Dacus	*tenebricus*	1
Dacus	*theophrastus*	5
Dacus	*transitorius*	3
Dacus	*triater*	1
Dacus	*umehi*	2
Dacus	*venetatus*	2
Dacus	*vertebratus*	21
Dacus	*woodi*	2
Dacus	*xanthopterus*	2
Dacus	*xanthopus*	2
Eristalinus	*aeneus*	1
Eristalinus	*albus*	4
Eristalinus	*arvorum*	1
Eristalinus	*cupreus*	1
Eristalinus	*dubiosa*	1
Eristalinus	*eclarus*	1
Eristalinus	*euzonus*	1
Eristalinus	*flaveolus*	1
Eristalinus	*fuscicornis*	4
Eristalinus	*goergeni*	2
Eristalinus	*gymnops*	1
Eristalinus	*lemmyi*	1
Eristalinus	*lineifacies*	2
Eristalinus	*macrops*	30
Eristalinus	*megametapodus*	1
Eristalinus	*modestus*	1
Eristalinus	*myiatropinus*	2
Eristalinus	*sexvittatus*	2
Eristalinus	*smaragdinus*	2
Eristalinus	sp.	10
Eristalinus	*tabanoides*	4
Eristalinus	*taeniops*	5
Eristalinus	*vicarians*	5
Eristalinus	*virescens*	1
Melanostoma	sp.	25

* **Note:** Female specimens of *Ceratitis rosa* and *C. quilicii* are morphologically indistinguishable using current diagnostic characters. As such, female individuals that could not be confidently assigned to either species were recorded as *C. quilicii/rosa*
