# Supplementary material for: Navigating uncertainty in museum workflows: genomic data mining and curation of the Diptera collections hosted at RMCA
Source: Biodivers Data J. 2025 Aug 12;13:e157274. doi: 10.3897/BDJ.13.e157274 (PMC12365672; doi:10.3897/BDJ.13.e157274)
Supplement: Supplementary material 2 — DNA extraction kits [file bdj-13-e157274-s002.pdf]

SM 2: DNA extraction kits used for the preliminary comparisons.

*according to the manufacturer’s instructions

DNA extraction kit	Spin column	Range DNA size	Expected DNA yield*	Cost / specimen (BTWI)
**DNeasy Blood and Tissue Kit (QIAGEN 69506)**	Dneasy spin column	100 bp-50 kb	6-30 µg	€ 5,20
**QIAamp Micro Kit (QIAGEN 56304)**	QIAamp MinElute column	<30 kb	<3 µg	€ 6,99
**QIAamp Mini Kit (QIAGEN 51306)**	QIAamp Mini spin column	<50 kb	4-30 µg	€ 5,45
**DNeasy Blood and Tissue Kit (QIAGEN 28006X4)**	MinElute column (MinElute PCR Purification Kit)	70 bp-4 kb	<5 µg	€ 8,49
