# Supplementary material for: Navigating uncertainty in museum workflows: genomic data mining and curation of the Diptera collections hosted at RMCA
Source: Biodivers Data J. 2025 Aug 12;13:e157274. doi: 10.3897/BDJ.13.e157274 (PMC12365672; doi:10.3897/BDJ.13.e157274)

SM 3: Total DNA yields from four DNA extraction protocols implemented on (A) whole-bodies, and (B) forelegs of Tephritidae and Syrphidae.

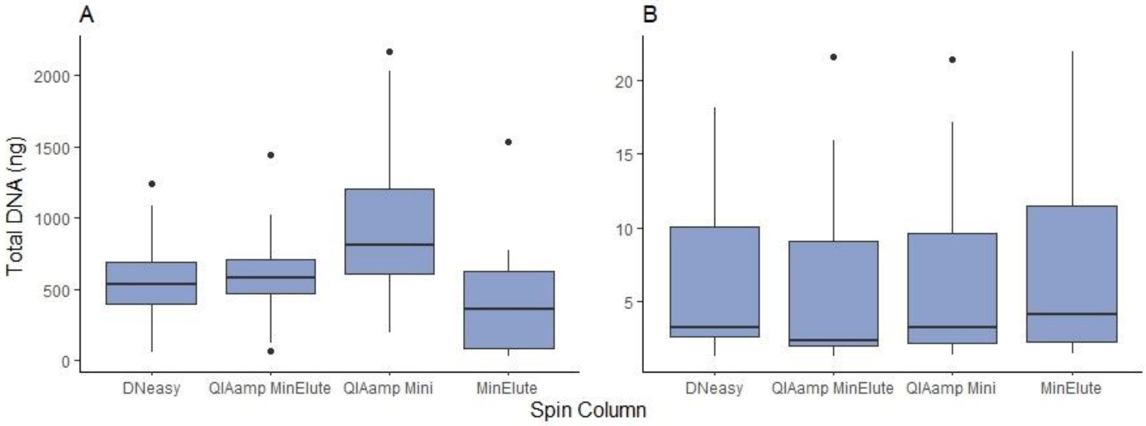

Supplement: Supplementary material 3 — DNA yields from DNA extraction protocols [file bdj-13-e157274-s003.pdf]
