# Supplementary material for: Navigating uncertainty in museum workflows: genomic data mining and curation of the Diptera collections hosted at RMCA
Source: Biodivers Data J. 2025 Aug 12;13:e157274. doi: 10.3897/BDJ.13.e157274 (PMC12365672; doi:10.3897/BDJ.13.e157274)
Supplement: Supplementary material 4 — ANCOVA [file bdj-13-e157274-s004.pdf]

SM 4: Analysis of Covariance (ANCOVA) of the general (LM) and generalised (GLM) linear models testing for the effects of voucher age (range 0-25 years) of Tephritidae and Syrphidae on six variables (total DNA/voucher, proportion of the DNA molarity of fragments shorter than 350 bp, proportions of 'pure' DNA measured through absorbance ratios 280/260 and 260/230nm, proportion of quality reads, proportion of aligned reads against GCA_029775095.1). P < 0.05 in bold. Df: degrees of freedom.

| | **Total DNA / voucher (LM)** | | | | | | | |
|---|---|---|---|---|---|---|---|---|
| | Tephritidae | | | | Syrphidae | | | |
| | *F*-value | *Mean Sq* | Df | *P*-value | *F*-value | *Mean Sq* | Df | *P*-value |
| Voucher age | 45.09 | 75.11 | 1 | **< 0.001** | 0.38 | 0.37 | 1 | 0.54 |
| Residuals | | 1.67 | 1174 | | | 85.96 | 88 | |
| | **Proportion short fragments (nmol/l) (LM)** | | | | | | | |
| | Tephritidae | | | | Syrphidae | | | |
| | *F*-value | *Mean Sq* | Df | *P*-value | *F*-value | *Mean Sq* | Df | *P*-value |
| Voucher age | 0.34 | 0.56 | 1 | 0.56 | 0.24 | 0.00029 | 1 | 0.62 |
| Residuals | | 1.67 | 738 | | | 0.10 | 88 | |
| | **A260/280 (GLM)** | | | | | | | |
| | Tephritidae | | | | Syrphidae | | | |
| | *LR* $\chi^2$-value | | Df | *P*-value | *LR* $\chi^2$-value | | Df | *P*-value |
| Voucher age | 53.04 | | 4 | **< 0.001** | 0.072 | | 1 | 0.79 |
| Residuals | | | 558 | | | | 80 | |
| | **A260/230 (GLM)** | | | | | | | |
| | Tephritidae | | | | Syrphidae | | | |
| | *LR* $\chi^2$-value | | Df | *P*-value | *LR* $\chi^2$-value | | Df | *P*-value |
| Voucher age | 59.92 | | 4 | **< 0.001** | 0.942 | | 1 | 0.33 |
| Residuals | | | 558 | | | | 80 | |
| | **Proportion Q > 30 reads (LM)** | | | | | | | |
| | Tephritidae | | | | Syrphidae | | | |
| | *F*-value | *Mean Sq* | Df | *P*-value | *F*-value | *Mean Sq* | Df | *P*-value |
| Voucher age | 11.98 | 25.03 | 1 | **< 0.001** | 0.53 | 0.46 | 1 | 0.47 |
| Residuals | | 2.09 | 1078 | | | 0.87 | 88 | |
| | **Proportion aligned reads (LM)** | | | | | | | |
| | Tephritidae | | | | Syrphidae | | | |
| | *F*-value | *Mean Sq* | Df | *P*-value | *F*-value | *Mean Sq* | Df | *P*-value |
| Voucher age | 38.80 | 74.06 | 1 | **< 0.001** | 0.87 | 0.0029 | 1 | 0.36 |
| Residuals | | 202.34 | 106 | | | 0.0033 | 26 | |
